# Prevalence and Molecular Characterization of FAdV‐4 Isolated in Fujian Province, Southeast China During 2023–2025

**DOI:** 10.1155/tbed/6535858

**Published:** 2026-08-30

**Authors:** Youlin Chen, Ziyue Wang, Dingping Bai, Zhen Chen, Cuiteng Chen, Xi Cai, Guanghua Fu, Chunhe Wan, Yu Huang, Chunhua Zhu

**Affiliations:** ^1^ Fujian Provincial Key Laboratory for Avian Diseases Control and Prevention, Institute of Animal Husbandry and Veterinary Medicine, Fujian Academy of Agricultural Sciences, Fuzhou 350013, China, faas.cn; ^2^ Fujian Key Laboratory of Traditional Chinese Veterinary Medicine and Animal Health, College of Animal Sciences, Fujian Agricultural and Forestry University, Fuzhou 350002, China, fjau.edu.cn

**Keywords:** fowl adenovirus serotype 4, genetic diversity, molecular characterization, prevalence, virus isolation

## Abstract

Fowl adenovirus (FAdV) serotype 4 (FAdV‐4) is a significant pathogen causing hepatitis–hydropericardium syndrome (HHS) in poultry, which can cause severe economic losses to the global poultry industry. Molecular epidemiological investigation of FAdV‐4 has not been conducted in Fujian province, southeast China. In this study, we collected 219 samples of liver exhibiting similar pathological features of hepatitis or hydropericardium syndrome (HPS)‐like clinicopathology changes from Fujian province during 2023–2025, which were analyzed using PCR assays. Further isolation of the virus was performed, and complete genomic sequences of the isolates were obtained through next‐generation sequencing technology. Genetic evolution analysis of the gene sequences was conducted to elucidate the prevalence, molecular characteristics, and genetic variations of FAdV‐4 in Fujian province. The results indicated that among the 219 clinical samples, 65 tested positive, yielding a positive rate of 29.68% (65/219). Positive samples from broilers were predominantly detected in the 21–120 days old, while those from layers were primarily concentrated in the 200–500 days old. The positive rate of Muscovy ducks infected with FAdV‐4 was 4.57%. Ultimately, seven strains of FAdV‐4 were isolated and designated as FJ08, FJ09, FJ20, FJ23, FJ33, FJ35, and FJ45. The preliminary challenge assay showed that all seven isolates were highly virulent in 7 days old SPF chickens, resulting in the death of all chickens within 2–3 days, leading to a mortality rate of 100%. Comparative analysis of whole genome sequences revealed that the isolated FAdV‐4 strains from Fujian province clustered within the major genetic lineage as the domestic FAdV‐4 reference strains (e.g., JSJ13, HLJFAd15, and HB1510 strains) and exhibited the highest identity with the FAdV‐4 BNC strain derived from black‐necked cranes in Yunnan province. Notably, the Hexon genes of seven isolated strains were divided into two major lineages based on phylogenetic analysis. The FJ08, FJ09, and FJ23 strains clustered in the major lineage as representative strains of FAdV‐4 from both domestic and international sources, whereas FJ20, FJ33, FJ35, and FJ45 strains formed an independent genetic lineage with the FAdV‐10 AHFY19 strain. The prevalence analysis revealed that FAdV‐4 has been circulating among commercial chickens, indigenous chicken breeds, and Muscovy ducks in Fujian province, southeast China, indicating that the FAdV‐4 strains in Fujian province possess the potential for cross–host transmission. Moreover, the age of the infected hosts (layers) has extended to 500 days, and their genomic characteristics exhibit genetic diversity.

## 1. Introduction

Fowl adenovirus (FAdV) is a prevalent pathogen in poultry, belonging to the genus *Aviadenovirus* within the family Adenoviridae, which induces various diseases in poultry, including inclusion body hepatitis (IBH), hydropericardium syndrome (HPS), and gizzard erosion [1]. Based on differences in group‐specific antigens, FAdVs are classified into groups I –III. Group I FAdVs are further divided into five species groups (FAdV‐A–E) and 12 serotypes (FAdV‐1–8a, FAdV‐8b–11). Among these, FAdV‐4, FAdV‐8a, FAdV‐8b, and FAdV‐11 are the predominant serotypes in China [2–4].

The global epidemiological landscape of FAdV exhibits significant geographical variation and temporal dynamics [5, 6]. Following the outbreaks of IBH in the United States in 1963 and HPS in Pakistan in 1983, FAdV has spread to over 20 countries worldwide, forming regional epidemic patterns characterized by specific serotypes. FAdV‐4‐dominated HPS remains highly prevalent in high‐temperature and high‐humidity regions of South Asia, the Middle East, and East Asia, while IBH, caused by FAdV‐8b and FAdV‐11, predominates in intensive farming areas of Europe and USA [7, 8]. China entered a high‐incidence period of FAdV‐4 in 2015, with northern regions accelerating interprovincial virus transmission through the cold chain transportation system associated with intensive white‐feathered broiler farming. In contrast, southern live poultry trading networks, particularly involving yellow‐feathered broilers, have increased the risk of serotype recombination. The clinical isolates from the northern provinces predominantly consist of FAdV‐4, which is primarily responsible for causing highly lethal HPS. Conversely, the eastern and southeastern coastal provinces, under the dual pressures of a warm and humid climate and high‐density farming, have experienced co‐circulation of multiple serotypes [9, 10]. The source of FAdV infection exhibits significant host diversity, and its transmission mode is closely related to the host species characteristics and the viral serotype. As a typical pan‐host pathogen, FAdV can infect a variety of poultry species, including chickens, turkeys, ducks, geese, pigeons, ostriches, peacocks, and black‐necked cranes; however, the susceptibility and excretion characteristics vary significantly among different species [1]. Among poultry, chickens, particularly broilers aged 3–6 weeks, are the primary hosts and exhibit high susceptibility to FAdV‐4 and FAdV‐8b. In contrast, duck‐derived isolates predominantly belong to species D serotypes (FAdV‐2, 3, 9, and 11), which have significantly lower pathogenicity compared to chicken‐derived FAdV‐4 [11].

Recent epidemiological surveillance has indicated that the host range of FAdV‐4 has expanded beyond traditional broiler chickens to include layer chickens, partridge chickens, and native chickens, with an increasing detection rate in waterfowl, such as ducks. This trend suggests an enhanced potential for cross‐species transmission [12, 13]. The epidemiological characteristics of FAdV‐4 infection in duck hosts are particularly complex. Wild FAdV‐4 strains isolated in China can cause a high mortality rate of 37% in duck flocks; however, duck‐derived strains induce only 10% mortality when reverse‐infecting SPF chickens and do not elicit clinical symptoms in ducks of the same age. This observation suggests that ducks may serve as natural reservoirs for FAdV‐4 [14].

The novel genotype FAdV‐4 has begun to spread across various regions of China since 2015 [10, 15, 16]. Fujian province serves as a significant hub for poultry farming and consumption, characterized by diverse farming methods and a wealth of high‐quality indigenous chicken breeds. Most farms employ ecological free‐range farming practices. Additionally, the region’s warm and humid climate, along with its developed water systems, facilitates the survival and transmission of viruses. Geographically, Fujian province, located in the southeastern coastal region, engages in frequent trade with major poultry‐producing areas. The circulation of poultry has increased the risk of epidemic introduction; certain farms are positioned in regions characterized by dense transportation networks or intensive farming practices, which may contribute to regional outbreaks. Currently, reports indicate cases of poultry infected with FAdV‐4 in Fujian province [10, 17]. However, there are limited reports detailing the complete genome of FAdV‐4 in Fujian province, and the molecular characteristics of the genome remain to be elucidated.

This study collected clinical samples exhibiting similar pathological features of hepatitis or HPS‐like clinicopathological changes from Fujian province during the years 2023–2025. Additionally, virus isolation was performed on samples that tested positive exclusively for FAdV‐4 infection. By combining whole‐genome sequencing and phylogenetic analysis of key genes such as DNA polymerase, Fiber1, Fiber2, and Hexon genes, we systematically analyzed the epidemiological status and genetic variation characteristics of FAdV‐4 in Fujian province, providing a crucial basis for the scientific prevention and control of FAdV‐4 disease in the region.

## 2. Materials and Methods

### 2.1. Samples Collection and Processing

From 2023–2025, we collected a total of 219 clinical samples from farms in eight cities of Fujian province (Fuzhou, Nanping, Ningde, Sanming, Putian, Longyan, Quanzhou, and Zhangzhou). The samples primarily originated from commercial broiler farms and layer farms, as well as indigenous chicken breeds, which presented with the characteristics of a HPS‐like clinicopathology. The collected samples were aseptically cut into small pieces and placed in 5 mL sterile EP tubes, with two 5 mm magnetic beads added and 1 mL sterile phosphate‐buffered saline (PBS) (Thermo, Suzhou, China), and the samples were ground into a homogenate at −20°C and 50 Hz using a low‐temperature high‐speed tissue grinder. After centrifugation at 7000 × *g* for 15 min, the supernatant was aliquoted into 2 mL of sterile EP tubes, and the samples were subjected to three freeze–thaw cycles before being stored at −80°C for future use.

### 2.2. PCR Assay

The DNA and RNA nucleic acids of the samples were extracted using a nucleic acid extraction kit from BioFLUX (BIOER, Hangzhou, China). All samples were subjected to etiological detection for viruses including FAdV‐4, ALV, CIAV, etcetera. The PCR amplification was performed in a total volume of 20 μL, consisting of 10 μL 2×Taq Plus Master Mix (Vazyme, Nanjing, China), 1 μL forward primer (1 μM), 1 μL reverse primer (1 μM), 2 μL template DNA, and 6 μL ddH_2_O. The thermal cycling conditions were set as follows: pre‐denaturation at 94°C for 5 min (1 cycle), followed by 30 cycles of denaturation at 94°C for 30 s, annealing at 64°C for 30 s (56°C for CIAV), and extension at 72°C for 1 min; with a final extension at 72°C for 10 min. A positive control was included during amplification. After amplification, a 1% agarose gel was prepared. A 10 μL aliquot of each PCR product and 5 μL of the DL2000 DNA Marker (Vazyme, Nanjing, China) were loaded into the wells, followed by electrophoresis at a constant voltage of 220 V for 25 min. The results were visualized and recorded using a gel imaging system.

### 2.3. Chicken Kidney Cells Culture

SPF embryos were purchased from Jinan SPAFAS Poultry Co., Ltd. To isolate kidney tissues from SPF chicken embryos at 18 days old, we begin by removing the kidney tissues and washing them three times with pre‐cooled PBS. Subsequently, the tissues were cut into small pieces and added 2 mL of 0.25% trypsin (Gibco, Paisley, UK), incubating at 37°C for five consecutive intervals of 2 min each. After incubation, the mixture was filtered to obtain a single‐cell suspension. To terminate the digestion, 4 mL of FBS (OriCell, New Zealand) was added and the solution was vortexed. The filtrate was centrifuged at 1200 × *g* for 3 min, discarding the supernatant. The cells were resuspended in 5 mL of DMEM (Thermo, Suzhou, China) complete medium containing 10% FBS and 1% penicillin/streptomycin, diluting the cell suspension to a density of 1 × 10^6^/mL before inoculating into 25 mL cell culture flasks for cultivation (37°C, 5% CO_2_). Once the primary kidney cells reach 70%–80% confluence, they were washed twice with serum‐free medium containing 1% penicillin/streptomycin (Gibco, New York, USA).

### 2.4. Virus Isolation and Culture

Fresh tissue samples infected solely with FAdV‐4 were selected for virus isolation. The lysate samples of the liver tissues were centrifuged at 8000× *g* for 30 min. The supernatant of the tissue lysis solution was then filtered through a 0.22 μm filter for sterilization before being inoculated into the monolayer primary kidney cells. In each 25 mL cell culture flask, 1 mL of sterile tissue lysate supernatant was inoculated. Following inoculation, the virus was incubated at 37°C in a 5% CO_2_ atmosphere for 2 h before discarding the supernatant. Subsequently, DMEM medium supplemented with 2% FBS and 1% penicillin/streptomycin was added. The cells were cultured for 3–5 days, or until approximately 80% of the cells exhibited pathological changes. The virus solution was freeze–thawed three times for use in the cultivation of the next generation of FAdV‐4, continuing the cultivation for six generations. The qPCR assay was used to detect the viral nucleic acid load of isolated strains.

### 2.5. Pathogenicity of the Isolated Strains in SPF Chickens

The 7 days old SPF chickens were obtained from Jinan SPAFAS Poultry Co., Ltd. and were housed in an isolator with a relative humidity of 40%–60% for rearing. A total of 135 7‐day‐old SPF chickens were divided into nine groups, each containing 15 chickens. The chickens were kept in isolators and monitored continuously for 14 days. The method and dosage for the viral challenge were adapted from the reference by Zhu et al. [4]. Chickens in the virus‐challenged groups received an intramuscular injection of 0.5 mL of the viral solution, while those in the NC group were injected with the same volume of PBS buffer. The GDMZ strain was used as the positive control. Following the viral challenge, the clinical symptoms of chickens were observed daily. Necropsies were conducted on moribund chickens to assess the pathological changes in their tissues and organs. Liver tissues were collected, cut into small pieces measuring approximately 0.5 cm × 0.5 cm × 0.3 cm, fixed in paraformaldehyde for 24 h, and prepared for HE staining analysis. The early pathological damages to the liver tissues caused by infection with the isolated strains were examined under an optical microscope.

### 2.6. Next‐Generation Sequencing

Seven strains of virus were isolated, and their genomic DNA was extracted and sent to Sangon Biotech (Sangon Biotech, Shanghai, China). The genome sequences of the FAdV‐4 virus were obtained through next‐generation sequencing. The concentration of the extracted genomic DNA was determined using a Qubit 4.0 fluorometer (Thermo, Q33226). The integrity of DNA was assessed via 1% agarose gel electrophoresis. The whole genomic DNA was randomly fragmented to an average size of 200−400 bp. The selected fragments underwent end repair, 3^′^ adenylation, adapter ligation, and PCR amplification. After purification using magnetic beads, the library was assessed for quality with a Qubit fluorometer, and its length was evaluated through 2% agarose gel electrophoresis. The qualified libraries were sequenced on the Illumina NovaSeq 6000 platform with 300 bp reads at Sangon Biotech (Shanghai, China). Following sequencing, raw reads were filtered using Trimmomatic (v0.36) to remove adapters and low‐quality reads, resulting in clean reads. Genome assembly was performed utilizing SPAdes (v3.15), and GapCloser (v1.11) was employed to fill the gaps.

### 2.7. Phylogenetic and Homology Analysis

A total of 24 sequences were retrieved from GenBank, comprising representative strains of various serotypes registered by the ICTV alongside selected domestic strains. Phylogenetic trees were constructed using MEGA 12 software, focusing on the full genome, DNA polymerase, Hexon gene, and Fiber gene, respectively. The neighbor‐joining (NJ) method was employed for phylogenetic analysis, utilizing a Bootstrap value of 1000 replicates to assess the evolutionary relationships between the isolated strains and reference strains. Homology analysis was conducted using the MegAlign module in DNAstar software.

## 3. Results

### 3.1. Clinical Samples Detection

**Figure 1:**
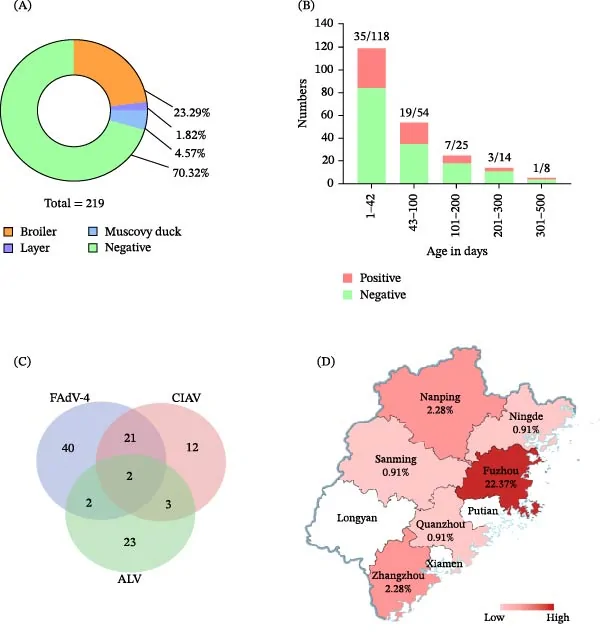
The prevalence of FAdV‐4 in Fujian province, southeast China. (A) The distribution of FAdV‐4 in different hosts in Fujian province. (B) The coverage range of different ages from collected samples. (C) Positive sample coinfection situation from collected samples. (D) The spatial distribution of positive samples in different regions.

The prevalence of FAdV‐4 in poultry within Fujian province is closely associated with the host species, age, and farming management practices. Most clinical samples originated from free‐range farms. A total of 219 samples were collected between 2023 and 2025, of which 65 tested positive, resulting in a positive rate of 29.68% (65/219). The positive rates for ALV and CIAV were 13.70% (30/219) and 17.35% (38/219), respectively. Chickens constituted the primary infected group, with 55 cases (25.11%), including 51 positive broilers (23.29%) and four positive layers (1.82%), along with 10 cases in Muscovy ducks (4.57%) (Figure 1A). Regarding age distribution, positive rates of FAdV‐4 varied across different age groups: 29.66% (35/118) in poultry aged ≤42 days, 35.19% (19/54) in those aged 43–100 days, 28.00% (7/25) in poultry aged 101–200 days, 21.43% (3/14) in those aged 201–300 days, and 20.00% (1/5) in poultry aged 301–500 days (Figure 1B). Coinfections were noted on the farms, with 23 cases involving coinfection with CIAV and four cases with ALV among the 65 FAdV‐4 positive samples, as well as two instances of triple infection with FAdV‐4, CIAV, and ALV (Figure 1C). The research indicated that the positive rate (22.37%) was the highest in Fuzhou city within Fujian province, followed by Zhangzhou and Nanping, both at 2.28%. In contrast, the positive rates of FAdV‐4 in Quanzhou, Ningde, and Sanming were comparatively lower (Figure 1D).

### 3.2. Virus Isolation and Culture

**Figure 2:**
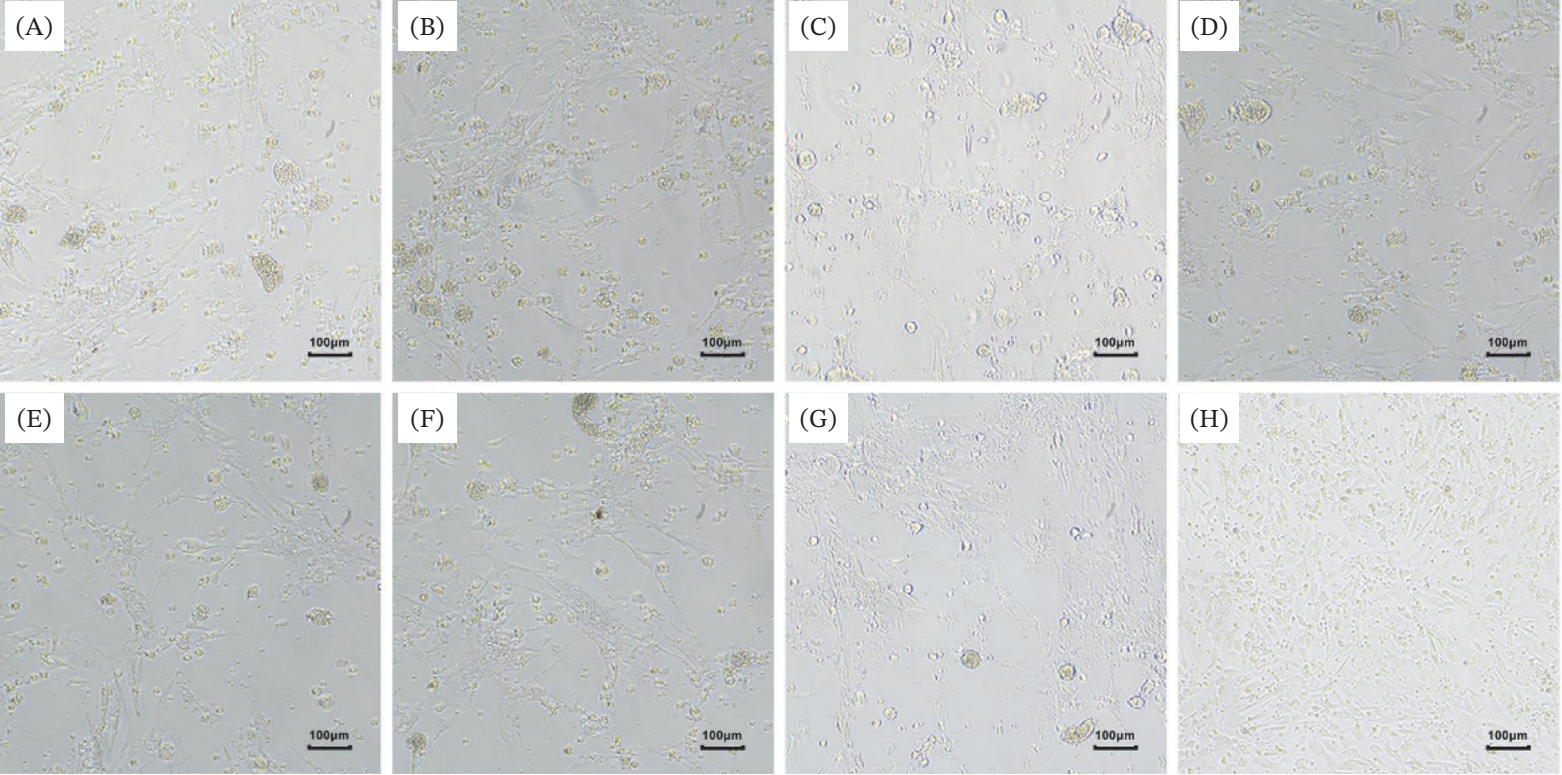
CEK cells were infected by FAdV‐4 strains. (A) FJ08 strain. (B) FJ09 strain. (C) FJ20 strain. (D) FJ23 strain. (E) FJ33 strain. (F) FJ35 strain. (G) FJ45 strain. (H) Normal control.

The supernatant of the grinding liquid from positive samples infected solely with FAdV‐4 was inoculated into primary chicken embryo kidney (CEK) cells for virus isolation, continuous passage, and cultivation. Ultimately, seven distinct FAdV‐4 strains were obtained and designated as FJ08, FJ09, FJ20, FJ23, FJ33, FJ35, and FJ45. After being passaged to the third generation, significant cytopathic effects were observed 48 h post‐inoculation with FAdV‐4. The CEK cells exhibited swelling, rounding, aggregation, and detachment, resulting in a disrupted mesh‐like pattern (Figure 2). In contrast, the normal CEK cells in the control group showed no significant changes. DAdV‐3 achieved stable proliferation after being passaged from the fifth to the sixth generation, with viral nucleic acid load ranging between 2 × 10^6^ and 5 × 10^6^ copies/μL.

### 3.3. Pathogenicity of the Isolated Strains in SPF Chickens

Following the challenge, all SPF chickens exhibited typical clinical symptoms, including listlessness, reduced appetite, and slightly closed eyes. By the second day post‐challenge, some chickens began to show signs of weakness in their legs, while others suddenly collapsed and died. On the second day post‐challenge, the mortality rate was highest in the challenged groups with the FJ09 strain, with all 15 chickens succumbing; 14 chickens died in the group infected with the FJ45 strain, while only one chicken died in each of the groups inoculated with the FJ08, FJ20, and FJ35 strains. A peak in mortality was observed at 3 days post‐challenge, with all chickens across each experimental group perishing, resulting in a mortality rate of 100% (Table 1). Notably, no deaths occurred in the PBS‐control group throughout the entire experimental period. These results indicate that the seven isolated FAdV‐4 strains are highly pathogenic to 7‐day‐old SPF chickens.

**Table 1:** Pathogenicity of the isolated strains in SPF chickens.

Strain	24 h	48 h	72 h	Total number of deaths	Mortality (%)
FJ08	0/15	1/15	14/15	15	100
FJ09	0/15	15/15	0/15	15	100
FJ20	0/15	1/15	14/15	15	100
FJ23	0/15	1/15	14/15	15	100
FJ30	0/15	1/15	14/15	15	100
FJ33	0/15	1/15	14/15	15	100
FJ45	0/15	14/15	1/15	15	100
GDMZ	0/15	1/15	10/15	11	73.33
PBS control	0/15	0/15	0/15	0	0

**Figure 3:**
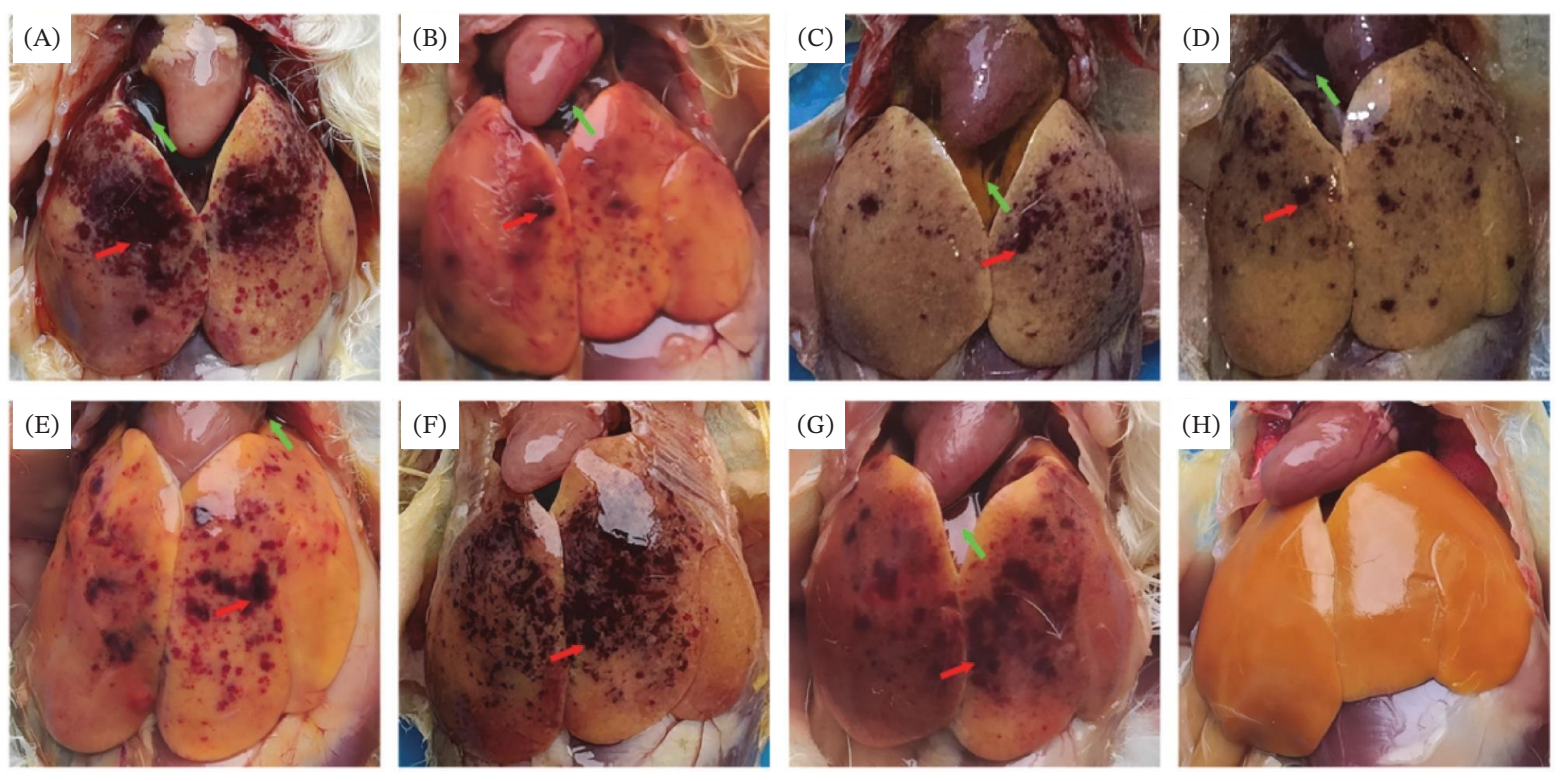
Observation of pathological changes of heart and liver tissues. (A) FJ08 strain. (B) FJ09 strain. (C) FJ20 strain. (D) FJ23 strain. (E) FJ33 strain. (F) FJ35 strain. (G) FJ45 strain. (H) Normal control. Yellow gel‐like exudate was observed within the pericardium (green arrow), alongside hemorrhagic spots and patches on the surface of the liver (red arrow).

During the autopsy of chickens, typical symptoms of hepatitis–HPS (HHS) were observed. A yellow, gel‐like exudate was present in the pericardium, and the liver tissue exhibited swelling and congestion. Upon palpation, the liver was found to be brittle and susceptible to cracking. The surface displayed diffuse pinpoint hemorrhages, with some areas merging into scattered bleeding spots. In contrast, the liver of chickens in the normal control group retained a normal red‐brown color, exhibited a firm texture, and had a smooth surface, without any lesions in color, texture, or surface (Figure 3).

### 3.4. Histopathology Analysis

**Figure 4:**
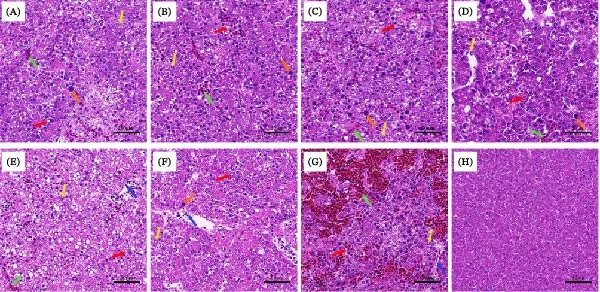
Histopathology observation of liver tissue sections. (A) FJ08 strain. (B) FJ09 strain. (C) FJ20 strain. (D) FJ23 strain. (E) FJ33 strain. (F) FJ35 strain. (G) FJ45 strain. (H) PBS group. Fragmentation or dissolution of the cell nucleus (yellow arrow). Fat vacuoles (red arrow). Accumulation of red blood cells within the hepatic sinusoids and portal areas (green arrow). Liver cell nucleus appears enlarged (orange arrow). Inflammatory cell infiltration (blue arrow).

The observation results of liver tissue sections revealed that the liver cells in the negative control group exhibited a normal physiological structure. In contrast, after infection with various FAdV‐4 strains in SPF chickens, a significant degree of fatty degeneration was observed in the liver tissues. Numerous round and transparent fat vacuoles appeared within the cytoplasm of the liver cells, with some vacuoles compressing the cell nucleus and displacing it toward the cell membrane. Small focal necrosis of liver cells was noted around the portal area, where the cell nuclei appeared fragmented or dissolved and the cytoplasm exhibited eosinophilic characteristics. Occasional infiltration of inflammatory cells was observed surrounding the necrotic foci, alongside an accumulation of red blood cells in the hepatic sinusoids and portal areas, where the vascular cavities were dilated and distorted (Figure 4).

### 3.5. Phylogenetic Analysis of the Complete Genome Sequences

**Figure 5:**
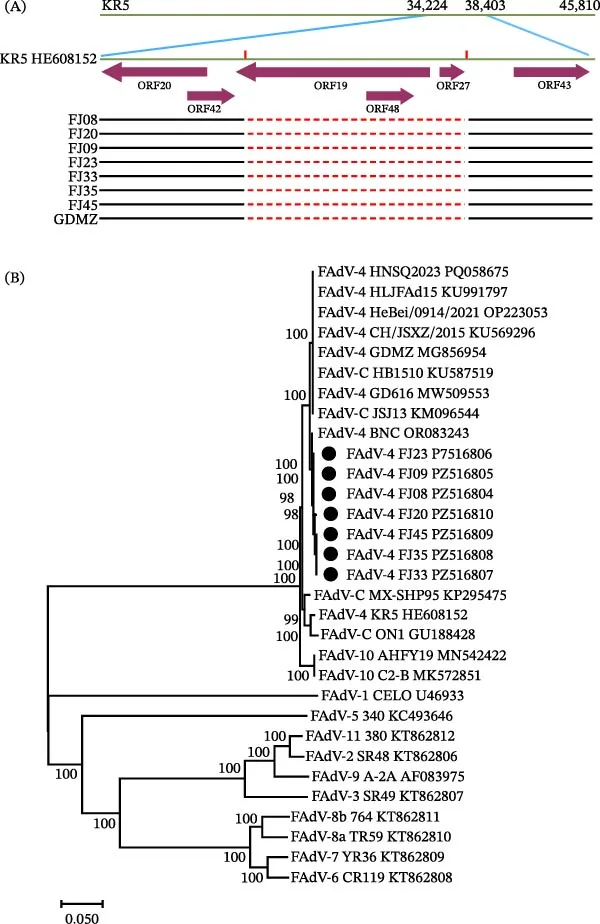
Genetic characteristics of FAdV‐4. (A) Genomic deletions in the isolated strains from Fujian province. The isolated Fujian strains displayed a significant gene deletion of 1966 bp at the locus of 35,413–37,378 bp when compared to foreign KR5 strains. (B) Phylogenetic tree of complete geneome sequences from isolated strains.

Further complete genomic sequences of the isolates were obtained using the next‐generation sequencing technology. These sequences were subsequently submitted to GenBank, with the following GenBank IDs: FJ08 (PZ516804), FJ09 (PZ516805), FJ20 (PZ516810), FJ23 (PZ516806), FJ33 (PZ516807), FJ35 (PZ516808), and FJ45 (PZ516809). The whole genome lengths ranging from 43.7 to 46.1 kb and GC content between 54% and 55%. Notably, the isolated Fujian strains displayed a significant gene deletion of 1966 bp at the locus of 35,413–37,378 bp when compared to the foreign KR5 (HE608152) and ON1 (GU188428) strains. This deletion resulted in the loss of reading frames for ORF19, ORF27, and ORF48 (Figure 5A).

The complete genomic sequences of the seven isolated strains were compared with the genomic sequences of 12 serotypes of FAdV registered in GenBank as well as selected domestic representative strains. A phylogenetic tree was constructed using MEGA 12 software. The homology of the domestic FAdV‐4 representative strains exhibited a gradient difference: the Fujian isolated strains showed the highest identity with the Yunnan BNC strain (OR083243, host: black‐necked crane) at 99.7%–100%, thereby forming a distinct evolutionary sub‐branch of FAdV‐4.The identity with domestic representative strains, such as the Hubei HB1510 strain, Jiangsu JSJ13 strain, and Guangdong GDMZ strain (including pigeons and blue‐necked quails as hosts), ranged from 99.4% to 99.6%; the identity with the Guangdong GD616 strain was 97.9%–98.1%; and the lowest identity was observed with the Anhui FAdV‐10 AHFY19 strain at 95.3%. The identity of the isolates with international FAdV‐4 reference strains was significantly lower than that of domestic strains. The Austrian KR5 strain (FAdV‐4) exhibited an identity of 95.5%–95.6%, and the C2‐B strain (FAdV‐10) showed an identity of 95.3%; among European and USA strains, the Mexican MX‐SHP95 strain had an identity of 95.8%–95.9%, and the Canadian ON1 strain showed an identity of 95.2%–95.3% (Figure 5B).

### 3.6. Phylogenetic Analysis of the DNA Polymerase Genes

**Figure 6:**
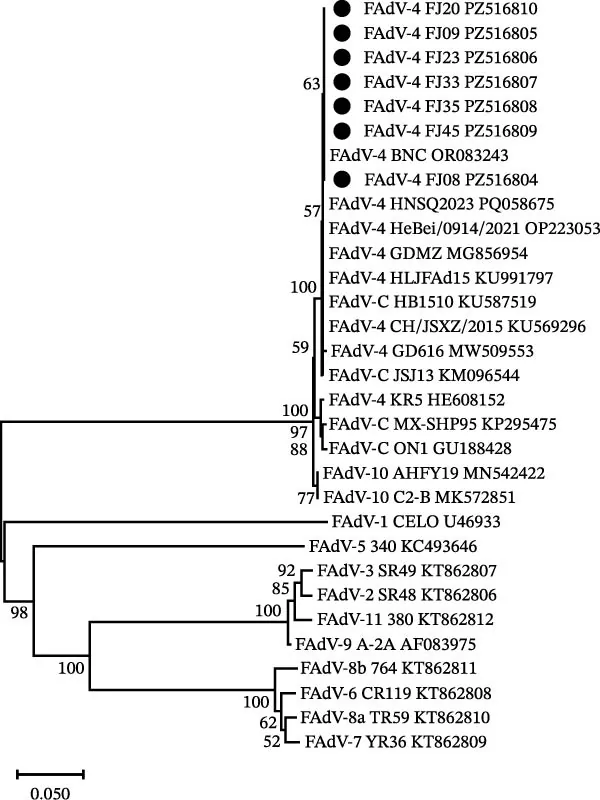
Phylogenetic tree of DNA polymerase genes from isolated strains. The scale bar represents the distance unit between sequence pairs.

DNA polymerase plays a critical role in catalyzing the synthesis of genomic DNA for viral progeny during the replication process, serving as the core enzyme for viral replication [18]. The genetic evolutionary tree indicates that the DNA polymerases from seven Fujian isolates form an independent branch alongside domestic representative strains (Figure 6). Notably, the DNA polymerases of these isolates exhibit 100% identity with domestic epidemic strains from 2015 to 2023, including the BNC strain from black‐necked cranes in Yunnan, the HeBei/0914/2021 strain from pigeons in Hebei, the GDMZ strain from layers in Guangdong, the HNSQ2023 strain from *Excalfactoria chinensis*, and the HB1510 strain from chicken in Hubei. Furthermore, they demonstrate 99.9% identity with the domestic strains JSJ13 from Jiangsu and GD616 from Guangdong, as well as 99.5% identity with the different serotype FAdV‐10 (AHFY19 strain from Anhui). Among international strains, the Fujian isolates show 99.3% identity with the KR5 strain from Austria and 99.2% identity with the ON1 strain from Canada and the MX‐SHP95 strain from Mexico.

### 3.7. Phylogenetic Analysis of the Fiber1 Genes

**Figure 7:**
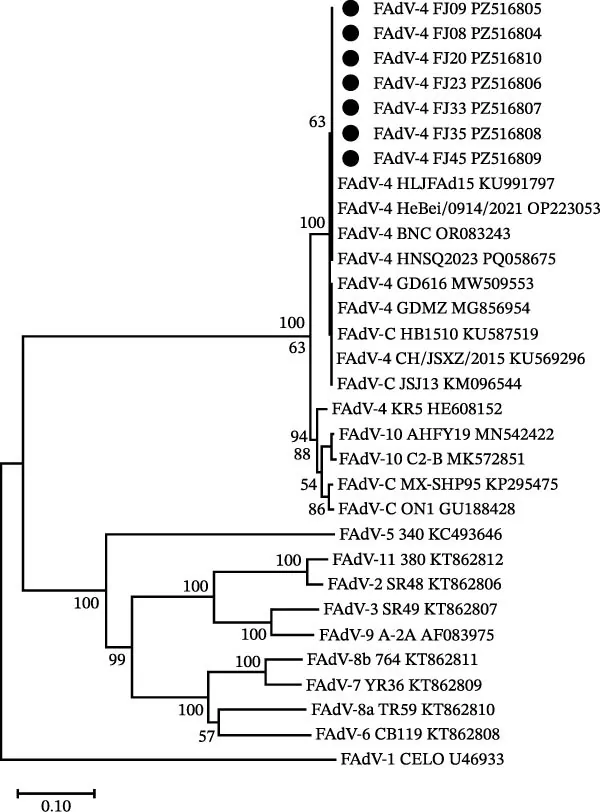
Phylogenetic tree of Fiber1 genes from isolated strains. The scale bar represents the distance unit between sequence pairs.

Fiber1 is a secondary fibrous protein located at the apex of the FAdV capsid, which collaborates with Fiber2 to facilitate host cell recognition and adsorption, thereby influencing the virus’s tissue specificity and infectivity [19]. The homology of the isolated strains from Fujian province with the chicken‐derived HLJFAd15 strain from Heilongjiang province, the black‐necked crane‐derived BNC strain from Yunnan province, the *Excalfactoria chinensis*‐derived HNSQ2023 strain from Henan province, and the pigeon‐derived HeBei/0914/2021 strain from Hebei province is 100%, indicating that they share a close evolutionary relationship. The protein sequence encoded by the Fiber1 gene of the Fujian isolate is entirely identical to that of these strains. However, the identity of the Fujian isolates with the JSJ13 strain, HB1510 strain, GDMZ strain, GD616 strain, and CH/JSXZ/2015 strain is 99.9%. The identity with international strains, including the Austria KR5 strain, the Canadian ON1 strain, and the Mexican MX‐SHP95 strain, ranges from 96.0% to 97.1%, which indicates a significant genetic distance from the FAdV‐4 branch to which the seven isolated strains belong. The identity between the Fujian isolates and the FAdV‐10 serotype strains (AHFY19 and C2‐B strain) was 96.4%–96.5% (Figure 7).

### 3.8. Phylogenetic Analysis of the Fiber2 Genes

**Figure 8:**
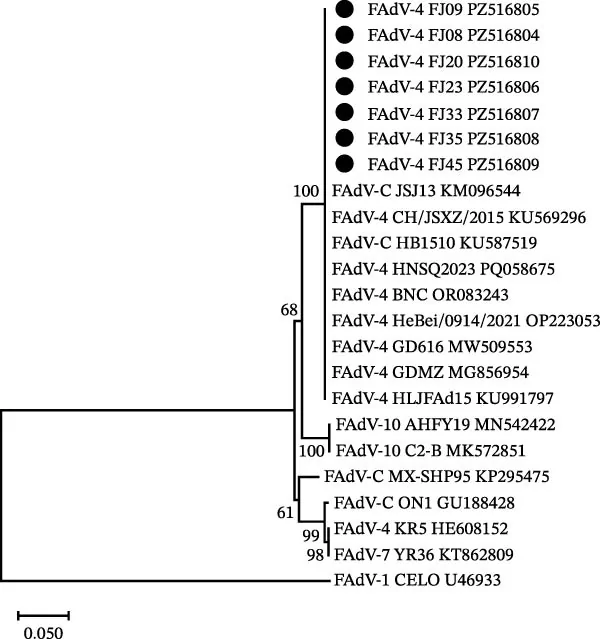
Phylogenetic tree of Fiber2 genes from isolated strains. The scale bar represents the distance unit between sequence pairs.

Fiber2 is a fibrous protein located on the surface of the FAdV capsid. It plays a crucial role in virus adsorption and invasion by recognizing host cell receptors, which determines tissue tropism and infection efficiency [20]. Genetic evolutionary analysis indicates that the Fiber2 gene is highly conserved across species. The Fiber2 gene sequences of the Fujian isolates cluster with domestic epidemic strains, including those from Jiangsu province (JSJ13 strain), Hubei province (HB1510 strain), Guangdong province (GDMZ strain), Heilongjiang province (HLJFAd15 strain), Yunnan province (BNC strain), Henan province (HNSQ2023 strain), and Hebei province (HeBei/0914/2021 strain), exhibiting an identity of 100%. In contrast, the Fujian isolates are positioned in a separate branch alongside foreign representative strains, such as the Mexican strain MX‐SHP95, the Austrian strain KR5, and the Canadian strain ON1. The identity with the Mexican strain MX‐SHP95, Austrian strain KR5, and Canadian strain ON1 ranges from 95.9% to 97.1%, while the identity with the FAdV‐10 strain AHFY19 from Anhui province and the C2‐B strain from USA is 97.1% (Figure 8).

The protein encoded by the Fiber2 gene of the Fujian isolate was compared with the Fiber2 amino acid sequences of domestic reference strains, including GDMZ, JSJ13, HB1510, and HLJFAd15. The analysis revealed that the Fiber2 protein exhibited high conservation, with an identity of 100%. In contrast, comparisons with foreign strains indicated differences in the amino acid positions of the Fiber2 protein. Specifically, there were 27 amino acid mutations between the Fujian isolates and the Austrian KR5 strain, 32 mutations between the Fujian isolates and the Canadian strain ON1, and 27 mutations between the Fujian isolates and the Mexican strain MX‐SHP95. Furthermore, compared to ON1 and MX‐SHP95, the Fujian isolates exhibited five amino acid insertions at positions 11−15, namely, Glu, Asn, Gly, Lys, and Pro amino acids.

### 3.9. Phylogenetic Analysis of the Hexon Genes

The Hexon gene is highly informative for elucidating the genotyping characteristics of FAdV [21]. Phylogenetic analysis revealed that the nucleotide sequences of the Hexon genes of the isolated strains from Fujian province were divided into two major branches. FJ08, FJ09, and FJ23 strains clustered in the same major branch as representative strains of FAdV‐4 from both domestic and international sources, whereas FJ20, FJ33, FJ35, and FJ45 strains formed an independent evolutionary lineage with the different FAdV‐10 serotype of the AHFY19 strain from Anhui province and JB422 strain from South Korea. Among these, FJ08, FJ09, and FJ23 strains formed one sub‐branch, exhibiting 100% identity with domestic epidemic strains (e.g., Jiangsu JSJ13, Hubei HB1510, and Guangdong GDMZ). In contrast, FJ20, FJ33, FJ35, and FJ45 clustered into another sub‐branch with the FAdV‐10 AHFY19 strain, demonstrating an identity of 98.1–98.4% with domestic epidemic strains (e.g., Jiangsu JSJ13, Hubei HB1510, and Guangdong GDMZ). Additionally, these isolates exhibited an identity of 97.8%–98.9% with foreign epidemic strains, including the Mexican strain MX‐SHP95 (KP295475), the USA C2‐B strain (MK572851), and the Canadian strain ON1 (GU188428).

**Figure 9:**
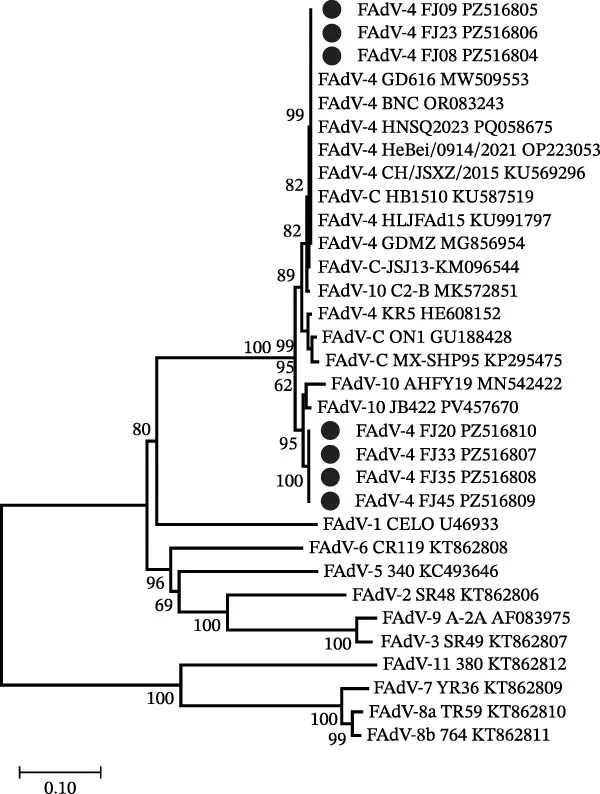
Phylogenetic tree of Hexon genes from isolated strains. The scale bar represents the distance unit between sequence pairs.

The proteins encoded by the Hexon gene of the isolates were compared with the Hexon amino acid sequences of commonly used reference strains in China (GDMZ, JSJ13, HB1510, HLJFAd15 strain, etc.). It was observed that strains FJ08, FJ09, and FJ23 were evolutionarily conserved and did not exhibit mutations (Table 2). In contrast, the FJ20, FJ33, FJ35, and FJ45 strains displayed a total of 21 mutations, including S162N, D166A, A197D, S208A, N211K, T240A, N243K, I263M, V264I, E269D, K394T, T400A, A401G, A402T, A408Q, N411G, S429N, S452N, I557V, S572T, and Q795E. These findings are consistent with the genetic evolution results of the Hexon genes (Figure 9).

**Table 2:** Mutation sites of amino acids in the Hexon protein of the isolated strains.

Strain	Amino acid site																		
	162	166	197	208	211	240	243	263	264	269	394	400–402	408	411	429	452	577	572	795
FJ08	S	D	A	S	N	T	N	I	V	E	K	TAA	A	N	S	S	I	S	Q
FJ09	S	D	A	S	N	T	N	I	V	E	K	TAA	A	N	S	S	I	S	Q
FJ20	N	A	D	A	K	A	K	M	I	D	T	AGT	Q	G	N	N	V	T	E
FJ23	S	D	A	S	N	T	N	I	V	E	K	TAA	A	N	S	S	I	S	Q
FJ33	N	A	D	A	K	A	K	M	I	D	T	AGT	Q	G	N	N	V	T	E
FJ35	N	A	D	A	K	A	K	M	I	D	T	AGT	Q	G	N	N	V	T	E
FJ45	N	A	D	A	K	A	K	M	I	D	T	AGT	Q	G	N	N	V	T	E
JSJ13	—	—	—	—	—	T	N	I	V	E	K	TAA	A	N	S	S	I	S	Q
HB1510	S	D	A	S	N	T	N	I	V	E	K	TAA	A	N	S	S	I	S	Q
KR5	S	D	A	S	N	A	E	M	I	E	K	TAA	A	N	S	S	I	S	E
ON1	S	D	A	S	N	A	E	M	I	E	K	TAA	A	N	S	S	I	S	Q
MX‐SHP95	S	D	A	S	N	A	E	M	I	E	K	TAQ	A	N	S	S	I	S	Q
GDMZ	S	D	A	S	N	T	N	I	V	E	K	TAA	A	N	S	S	I	S	Q
HLJFAd15	S	D	A	S	N	T	N	I	V	E	K	TAA	A	N	S	S	I	S	Q
BNC	S	D	A	S	N	T	N	I	V	E	K	TAA	A	N	S	S	I	S	Q
GD616	S	D	A	S	N	T	N	I	V	E	K	TAA	A	N	S	S	I	S	Q
CH/JSXZ/2015	S	D	A	S	N	T	N	I	V	E	K	TAA	A	N	S	S	I	S	Q
HNSQ2023	S	D	A	S	N	T	N	I	V	E	K	TAA	A	N	S	S	I	S	Q
HeBei/0914/2021	S	D	A	S	N	T	N	I	V	E	K	TAA	A	N	S	S	I	S	Q

## 4. Discussion

Since 2015, regional outbreaks of novel genotype FAdV‐4 have been reported in South, East, and Southwest China. Post‐mortem examinations reveal a yellow gelatinous exudate in the pericardium, along with hepatomegaly characterized by congestion and necrotic foci. The prolonged course of the disease often leads to fibrinous hepatitis, resulting in substantial economic losses for both broiler and layer farming [22, 23]. However, there are few studies addressing the prevalence of FAdV‐4 in Fujian province, making the conduction of epidemiological research and molecular characterization analysis of FAdV‐4 in this region of great significance.

This study collected clinical samples of hepatitis and HPS‐like pathological conditions from various regions of Fujian province between 2023 and 2025, totaling 219 samples for PCR testing to determine the FAdV‐4 infection. The positive rate was 29.68% (65/219), which is significantly higher than the positive rates of other viruses, such as ALV (13.70%) and CIAV (17.35%). From the perspective of host distribution, the detection rate of FAdV‐4 in chicken samples was the highest. Among these, positive samples from broilers accounted for a substantial 78.46% (51/65), indicating a relatively high infection rate in this group. The age of infection primarily ranged from 21 to 120 days. Notably, the older broilers were predominantly indigenous chicken breeds from Fujian province, including Hetian chicken, Minqing booted chicken, and Dehua black‐bone chicken. Preliminary statistics indicate that this study identified 11 positive samples from indigenous broilers in Fujian province, suggesting that FAdV‐4 may be disseminating among various local indigenous broilers. Numerous previous reports have documented the detection of FAdV‐4 in broilers [10, 24, 25]. This may be attributed to high‐density breeding conditions and the short growth cycle of broilers, combined with the incomplete development of immune organs, which result in relatively weak natural resistance to the virus. Notably, the positive samples detected in this study among layers were from poultry aged 200–500 days. This represents the first report of layers being infected at 500 days, indicating that there remains a risk of FAdV‐4 infection in layer farms in Fujian province concerning the transmission routes and immune barriers. This situation necessitates enhanced biosecurity control measures. Furthermore, the positive rate of FAdV‐4 infection in ducks was 4.57% (10/219), suggesting that potential cross‐species transmission characteristics of FAdV‐4 also exist in Fujian province, and the immune strategies in duck farms are insufficient to combat this virus [12, 26, 27].

The analysis of coinfection situations in 65 cases positive for FAdV‐4 from livestock farms in Fujian province revealed an overall coinfection rate of 38.46% (25/65). Among these cases, CIAV was identified as the primary coinfecting pathogen, with a double infection rate of CIAV and FAdV‐4 accounting for 35.38% (23/65). The coinfection rate between FAdV‐4 and CIAV was the highest observed. Previous studies have indicated that CIAV infection can exacerbate the clinical symptoms of FAdV [13, 28, 29]. This phenomenon may be attributed to the immunosuppressive properties of the VP3 protein of CIAV, which inhibits host T‐cell activity and disrupts thymus structure, thereby impairing the host’s immune response to FAdV‐4 and creating an immune window for FAdV‐4 invasion [30]. Additionally, the Hexon protein of FAdV‐4 triggers an inflammatory cytokine storm by activating the NF‐κB pathway, leading to the overexpression of pro‐inflammatory factors such as IL‐6 and TNF‐α. The synergistic effects of these factors may exacerbate hepatocyte apoptosis and the clinical progression of HPS [31].

Whole‐genome evolutionary analysis indicates that the seven Fujian isolates form an independent lineage within the FAdV‐C subgroup, closely related to the reference strains. These Fujian isolates exhibit the highest identity with the Yunnan BNC strain, which originates from black‐necked cranes, suggesting a potential for cross‐species transmission among various poultry hosts. Notably, the identity between the Fujian isolates and the FAdV‐10 strains (AHFY19 and C2‐B) is significantly lower than that observed with the FAdV‐4 strains of the same serotype, aligning with the observed differences in serological genetic distance. DNA polymerase, as the core enzyme for FAdV‐4 replication, its genetic characteristics directly reflect the virus’s replication potential and evolutionary context. DNA polymerase, as the core enzyme for FAdV‐4 replication, directly reflects the virus’s replication potential and evolutionary context through its genetic characteristics. The DNA polymerase sequences of the seven isolates from Fujian formed a branch with the reference strains of FAdV‐C in the phylogenetic tree. These Fujian‐isolated strains were closely clustered with a short genetic distance, confirming their classification as “serotype 4 of the C subgroup” and revealing a common evolutionary origin or recent regional transmission association among the Fujian isolates. Homology analysis demonstrated that the DNA polymerase of the isolates exhibited significantly higher identity (99.9%–100%) with domestic FAdV‐4 reference strains compared to international strains and different serotype FAdV‐10 (99.2%–99.5%). This finding indicates that domestic FAdV‐4 strains maintain high conservation in the DNA polymerase gene sequence, possibly due to a common evolutionary origin or frequent gene exchange among domestic strains. The low identity with foreign strains may be attributed to geographical factors influencing the evolution of FAdV‐4. Transmission barriers between different regions, along with variations in host populations and environmental selection pressures, have contributed to the formation of independent evolutionary lineages at the genetic level. The isolated strains demonstrate a nucleotide deletion of 1966 bp, which results in the absence of the open reading frames ORF19, ORF27, and ORF48. This finding aligns with the molecular characteristics associated with the domestic novel FAdV‐4 [32, 33]. The Fiber1 domain of FAdV‐4 plays a significant role in mediating infection and immune response [34, 35]. The pathogenicity of FAdV‐4 is closely related to the Fiber2 protein [33]. During the process of viral infection, the Fiber2 protein can directly bind to the receptors on the host cell membrane, thereby influencing the pathogenicity of the virus [36]. The study of the interaction domains of Fiber2 has shown that certain domains significantly affect replication and pathogenicity, although Fiber2 itself is not the main determinant of virulence [20]. The amino acid sequence of Fiber2 isolated from Fujian province was 100% identical to other prevalent strains in China, showing a highly conserved evolutionary pattern.

Notably, the four Fujian isolates (FJ20, FJ33, FJ35, and FJ45) and the JB422 strain from South Korea, along with the AHFY19 strain from Anhui province, cluster within a distinct lineage. In contrast, the C2‐B strain from USA clusters on the same lineage as FJ08, FJ09, and FJ23 strains as well as domestic representative strains. This finding not only highlights the genetic diversity of FAdV‐4 isolates in Fujian province but also indicates potential genetic characteristics shared among Asian FAdV‐10 isolates. However, only two complete gene sequences of FAdV‐10 are available in GenBank: the AHFY19 strain from Anhui province, China and the C2‐B strain from USA, while the genome sequence of the FAdV‐10 JB422 strain from South Korea is incomplete; however, it does contain a complete sequence of the Hexon gene. Although FAdV‐10‐like Hexon lineage (including FJ20, FJ33, FJ35, and FJ45 strains) exhibited serotype divergence, there is currently a lack of related analysis concerning the genomic characteristics of the FAdV‐10 Hexon gene, which requires further investigation in the future.

The mutation of the Hexon protein in FAdV‐4 has been established as a critical factor influencing the virus’s pathogenicity. Numerous studies have highlighted the significance of specific amino acid residues within the hexamer protein, particularly the 188th residue, which is pivotal in determining the virus’s virulence [37, 38]. Wang et al. [38] employed point mutations to alter 13 amino acid difference sites in the Hexon gene of both virulent and nonpathogenic strains of FAdV‐4, confirming that the 188th amino acid of Hexon is decisive for FAdV‐4 virulence. Generally, virulent strains possess R188, while nonpathogenic strains predominantly exhibit I188. Three‐dimensional spatial structure analysis suggests that the R188 mutation may induce a conformational change in the L1 ring of the Hexon protein, thereby influencing the interaction between the virus and host cells or facilitating immune evasion, which in turn reduces virulence. However, in the case of the nonpathogenic strain ON1, the mutation from Ile (I188) to Arg (I188R) amino acid did not enhance virulence; it resulted solely in IBH without causing pericardial effusion syndrome (HHS). This observation indicates that the manifestation of HHS necessitates the cooperation of additional viral factors [38]. In this study, the position 188 amino acid of Hexon protein of the two foreign nonpathogenic strains, KR5 and ON1, was identified as isoleucine, whereas that site of the seven isolated strains was arginine. Preliminary challenge experiments have confirmed that 7 day old SPF chickens infected with these strains exhibit not only pathological changes, such as hepatitis or HPS‐like clinicopathological changes, but also a 100% mortality rate (20/20). This suggests that the seven strains isolated in Fujian province exhibited high virulence. Interestingly, this study indicates that the strains FJ20, FJ33, FJ35, and FJ45, isolated from Fujian province, independently cluster into a distinct minor branch with different FAdV‐10 serotype of the AHFY19 strain and JB422 strain from South Korea. Amino acid sequence alignment analysis revealed that these four strains (FJ20, FJ33, FJ35, and FJ45) exhibited 21 mutations when compared to the other three strains (FJ08, FJ09, and FJ23). The outer capsid side of the Hexon protein monomer contains three loops, namely, L1, L2, and L4, which are responsible for antibody binding [19, 38]. L1 represents the hypervariable regions (HVRs 1–4) and is commonly used for FAdV typing [19, 39]. Among the 21 mutation sites identified in this study, 10 mutations (S162N, D166A, A197D, S208A, N211K, T240A, N243K, I263M, V264I, and E269D) are located within the Hexon L1 hypervariable regions. Specifically, the mutations S162N and D166A are situated in the core region of HVR1, potentially interfering with antibody binding [40]. Analysis of the three‐dimensional conformation of the Hexon protein reveals that mutations (A197D, S208A, and N211K) in HVR2 are adjacent to the conserved cross‐neutralizing site ^213^AYGAYVK^219^ [41, 42]. Mutations at these sites may alter the antigenic properties of FAdV‐4, resulting in immune escape and changes in the FAdV serotype. Therefore, it is essential to construct site‐directed mutant strains using reverse genetics techniques to determine the effects of these mutations on the biological characteristics and pathogenicity of the strains. In this study, the capacity of these strains to induce severe disease across various tissues underscores their high virulence potential. However, pathogenicity is influenced by factors such as the age of infection and the dosage of FAdV‐4 strains. The molecular pathogenic mechanisms among the four strains (FJ20, FJ33, FJ35, and FJ45) in comparison to those among the three strains (FJ08, FJ09, and FJ23) require further investigation.

## 5. Conclusion

In summary, this study reveals the prevalence and molecular characteristics of FAdV‐4 in Fujian province. The epidemiological data indicate that broiler chickens are the primary susceptible hosts of FAdV‐4, accounting for 23.29% (51/219). Additionally, the infection has been observed in layers up to 500 days of age. Furthermore, the positive rate of 4.57% for FAdV‐4 in Muscovy ducks suggests a potential risk for cross‐host transmission of FAdV‐4. Phylogenetic analysis demonstrates that the FAdV‐4 strain prevalent in Fujian province shows significant similarity to other domestic strains. The strains isolated in Fujian province exhibit high nucleotide identity with the FAdV‐4 BNC strain derived from black‐necked cranes in Yunnan province, as well as with the Hubei HB1510 strain, the Jiangsu JSJ13 strain, the Guangdong GDMZ strain from chickens, the HNSQ2023 strain from *Excalfactoria chinensis* in Henan province, and the HeBei/0914/2021 strain from pigeons in Hebei. These suggest that the strains prevalent in Fujian province may have originated from other hosts or provinces in China. While the overall evolution of these strains is conservative, certain genetic differences are observed in specific genes. Notably, the FJ20, FJ33, FJ35, and FJ45 strains have formed an independent genetic lineage with the FAdV‐10 AHFY19 strain, likely due to genetic adaptive changes that have resulted in variations in the Hexon gene sequences of FAdV‐4 strains in Fujian province. The Fujian isolates of FAdV‐4 exhibit genetic diversity and high virulence. These research findings enrich the epidemiological data in southeast China and provide a crucial scientific basis for formulating prevention and control strategies as well as for selecting vaccines for FAdV‐4 in Fujian province.

## Author Contributions

**Chunhua Zhu**: conceptualization, methodology, project administration, formal analysis, funding acquisition, writing – original draft, review and editing. **Youlin Chen**: methodology, software, data curation, formal analysis, visualization, writing – original draft. **Ziyue Wang**: methodology, formal analysis, writing – original draft. **Dingping Bai**: formal analysis writing – original draft. **Zhen Chen**, **Cuiteng Chen**, **Xi Cai, and Guanghua Fu**: methodology, formal analysis. **Chunhe Wan**: funding acquisition. **Yu Huang**: supervision, review and editing.

## Funding

This work was supported by the Fujian Provincial Public Research Institute of Fundamental Research (Grants 2025R1071 and 2025R10240007), the China Agriculture Research System (Grant CARS‐41‐18), and the Research and Technology Program of the Fujian Academy of Agricultural Sciences (Grant DWHZ2026‐04).

## Disclosure

All the authors have contributed to the manuscript and approved the submitted version.

## Ethics Statement

The chicken challenge assay and procedure were approved by the Research Ethics Committee of the Institute of Animal Husbandry and Veterinary Science of Fujian Academy of Agricultural Sciences (Approval Number MYLISC2025‐008).

## Conflicts of Interest

The authors declare no conflicts of interest.

## Data Availability

The data that support the findings of this study are available upon reasonable request. The data are not publicly available due to ethical considerations.
